# Keep a level head to know the way ahead: How rodents travel on inclined surfaces?

**DOI:** 10.1016/j.isci.2022.104424

**Published:** 2022-05-18

**Authors:** Zohar Hagbi, Elad Segev, David Eilam

**Affiliations:** 1 School of Zoology, George S. Wise Faculty of Life Sciences, Tel-Aviv University, Israel; 2 Department of Applied Mathematics, Holon Institute of Technology, Holon, Israel

**Keywords:** Biological sciences, Behavioral neuroscience, Cognitive neuroscience

## Abstract

Animals traveling on a horizontal surface stabilize their head in relation to the substrate in order to gather spatial information and orient. What, however, do they do when traveling on an incline? We examined how three rodent species differing in motor abilities and habitats explore a platform tilted at 0–90°, hypothesizing that they would attempt to maintain bilateral vestibular cues. We found that traveling up or down was mainly straight vertically rather than diagonally, which results in identical bilateral vestibular cues. This was also achieved when traveling horizontally through rotating the head to parallel the horizontal plane. Traveling diagonally up or down was avoided, perhaps due to different bilateral vestibular cues that could hinder orientation. Accordingly, we suggest that maintaining identical bilateral cues is an orientational necessity that overrides differences in motor abilities and habitats, and that this necessity is a general characteristic of animals in motion.

## Highlights

• Three rodent species were tested on a platform inclined at 0°–90°
• Increased inclination results in traveling straight vertically or horizontally
• Both these shapes of trajectories feature a horizontal leveled head
• We suggest that such posture is required for spatial orientation when in motion

## Graphical abstract

**Figure:**
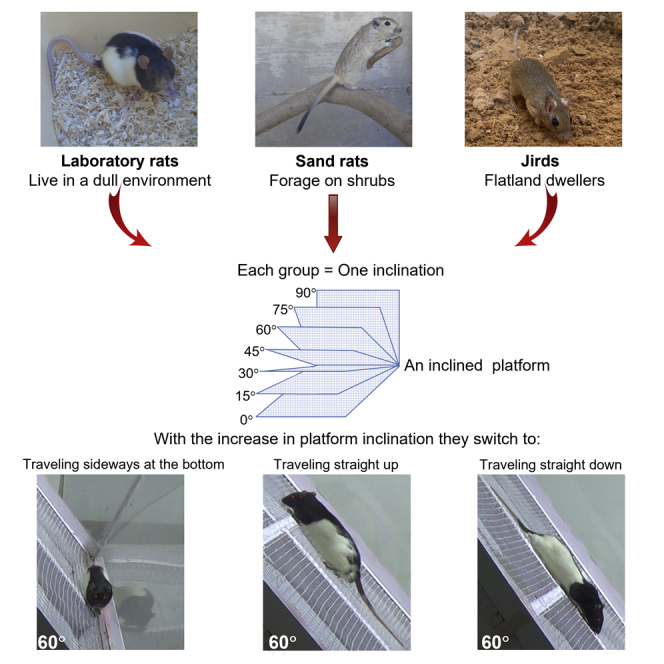

## Introduction

When in motion, humans and other animals tend to stabilize their head in relation to the horizontal plane. This stabilization seems essential for spatial orientation and for gathering spatial information, a process that depends on both external cues (e.g. vision) and internal cues (e.g. proprioception). These cues mainly rely on input from the vestibular and visual systems (Israel and Warren, 2005; MacNeilage et al., 2008). Vestibular signals have a central role since the visual system alone cannot compensate for their absence, and therefore, vestibular signals are required for effective spatial orientation (Banovetz et al., 2021; Yoder and Taube, 2014). Nevertheless, a study in rats suggested that vision can compensate for loss of vestibular information for some types of navigation (Wallace et al., 2002). Still, no visual compensation was revealed in humans with chronic bilateral vestibular loss, who exhibited navigation deficiency when tested in a virtual navigation task (Brandt et al., 2005). In mice with vestibular damage, the organization of exploratory behavior was disrupted, and access to visual cues did not help to balance the deficiency in vestibular information (Banovetz et al., 2021). The vestibular system is also essential for the activity of place cells and head direction cells, and a disfunction in these cells may affect spatial representation (Harvey et al., 2018; Russell et al., 2003; Valerio and Taube, 2016; Yoder and Taube, 2014). Indeed, temporary inactivation of the vestibular system leads to firing disruption in place and head direction cells (Stackman et al., 2002). Similarly, the activity of head direction cells was severely affected in rats that traveled upside-down (Valerio et al., 2010), preventing them from establishing a mental spatial representation (“map”) following repeated exposures to the same environment. Instead, in every exposure, they had to search anew for the target (Stackman et al., 2002; for a review:Valerio et al., 2010). By combining head stabilization with the necessity for vestibular cues, we argue here that when traveling in a three-dimensional environment it is crucial for animals to maintain identical bilateral vestibular cues. Accordingly, animals will attempt to maintain a leveled head.

The open-field is one of the most used apparatuses in spatial behavior research (Gould et al., 2009; Stanford, 2007; Walsh and Cummins, 1976). The spatiotemporal structure of open-field behavior and its correlated neural mechanisms have been thoroughly studied and reviewed (Eilam, 2014; Eilam and Golani, 1989; Thompson et al., 2018). However, those studies referred to behavior in a horizontal two-dimensional environment. Several studies in a three-dimensional lattice maze revealed that rats treat the horizontal and the vertical planes separately, exploring one level horizontally before exploring another horizontal level (Grobéty and Schenk, 1992; Jovalekic et al., 2011), or displaying a preference to remain at the bottom of the lattice maze (Jedidi-Ayoub et al., 2021). Additional studies have been conducted in other three-dimensional apparatus, including leveled pyramids (ziggurats; Faraji et al., 2010; Hagbi et al., 2020) and a set of six levels of bricks on which the rodents had to ascend or descend in order to progress (Gielman et al., 2020). The findings from these studies suggested that despite the three-dimensional structure of the apparatus, the rodents were able to preserve the structure of their spatiotemporal behavior similarly to in a two-dimensional open-field since they were able to maintain a horizontal body posture most of the time.

Here, we tested three rodent species that dwell in different habitats: laboratory rats, which are the most common subject in studying spatial behavior; fat sand rats (*Psammomys obesus*), which forage on desert shrubs and are therefore used to traveling in the vertical domain; and Tristram’s jirds (*Meriones tristrami*), which dwell in flatlands and are expected to have little experience in traveling vertically. In light of the above studies, we hypothesized that these rodents would attempt to maintain bilateral vestibular cues when traveling. To test this, we used a simple open-field that was tilted at various inclinations between 0° and 90°. Traveling on such an inclined surface forced the rodents to divert from their natural horizontal posture, as in the above-noted studies. Such diversion was expected to interfere with the bilateral vestibular cues that characterize traveling on a horizontal plane and reveal how animals respond when encountering such interference.

## Results

### Experiment 1

Here, we tested seven groups (n = 10 ea.) of each of the three rodent species. Each group was tested in a 1 × 1 m open-field with wire-mesh floor that provides grip, and with transparent Plexiglas walls that prevent falls. For each group, the open-field was tilted to one of the following inclinations: 0°, 15°, 30°, 45°, 60°, 75°, and 90° (see STAR Methods for Experiment 1 and Figure S1A in supplementary information 1). Each rodent was tested only once in only one inclination, and its behavior was video-recorded for 30 min.

#### Activity decreased with the increase in open-field inclination

Table 1 depicts the distance traveled by the three species in each open-field inclination. Factorial ANOVA with two factors, species and inclination, revealed that the total traveled distance decreased with the increase in open-field inclination (F_6,189_ = 7.22; p < 0.0001; η_p_^2^ = 0.1864); that there was no significant difference in traveled distance between the three species (F_2,189_ = 0.24; p = 0.7834; η_p_^2^ = 0.0026); and no interaction of species x inclination (F_12,189_ = 1.75; p = 0.0597; η_p_^2^ = 0.0999). Rats, sand rats, and jirds, all reduced their traveled distance with the increase in open-field inclination.

**Table 1:** Decrease in activity with the increase in open-field inclination

	Rats	Sand rat	Jirds
0°	94.9 ± 8.8	98.3 ± 13.5	107.8 ± 17.8
15°	80.0 ± 5.6	72.9 ± 9.7	67.2 ± 14.4
30°	93.8 ± 4.2	71.6 ± 13.0	81.3 ± 7.5
45°	77.5 ± 4.1	91.6 ± 16.1	70.1 ± 11.5
60°	56.2 ± 6.4	81.4 ± 13.2	59.9 ± 11.2
75°	**38.6 ± 6.6**^a^	71.1 ± 9.2	**54.5 ± 10.4**^a^
90°	57.4 ± 4.3	**37.8 ± 9.6**^a^	75.4 ± 14.4

Results of factorial ANOVA		
Effect of inclination	F6,126 = 9.71	**p < 0.0001**
Effect of species	F_2,126_ = 0.22	p = 0.8055
Interaction of inclination x species	F_12,126_ = 1.55	p = 0.1149

Mean ± SEM of the distance traveled (m.) throughout the arena for each inclination.

a In each species, indicates a significant difference compared to 0⁰ slope in Tukey HSD test (also depicted in bold).

#### Traveling mainly horizontally or vertically (straight up-down)

**Figure 1:**
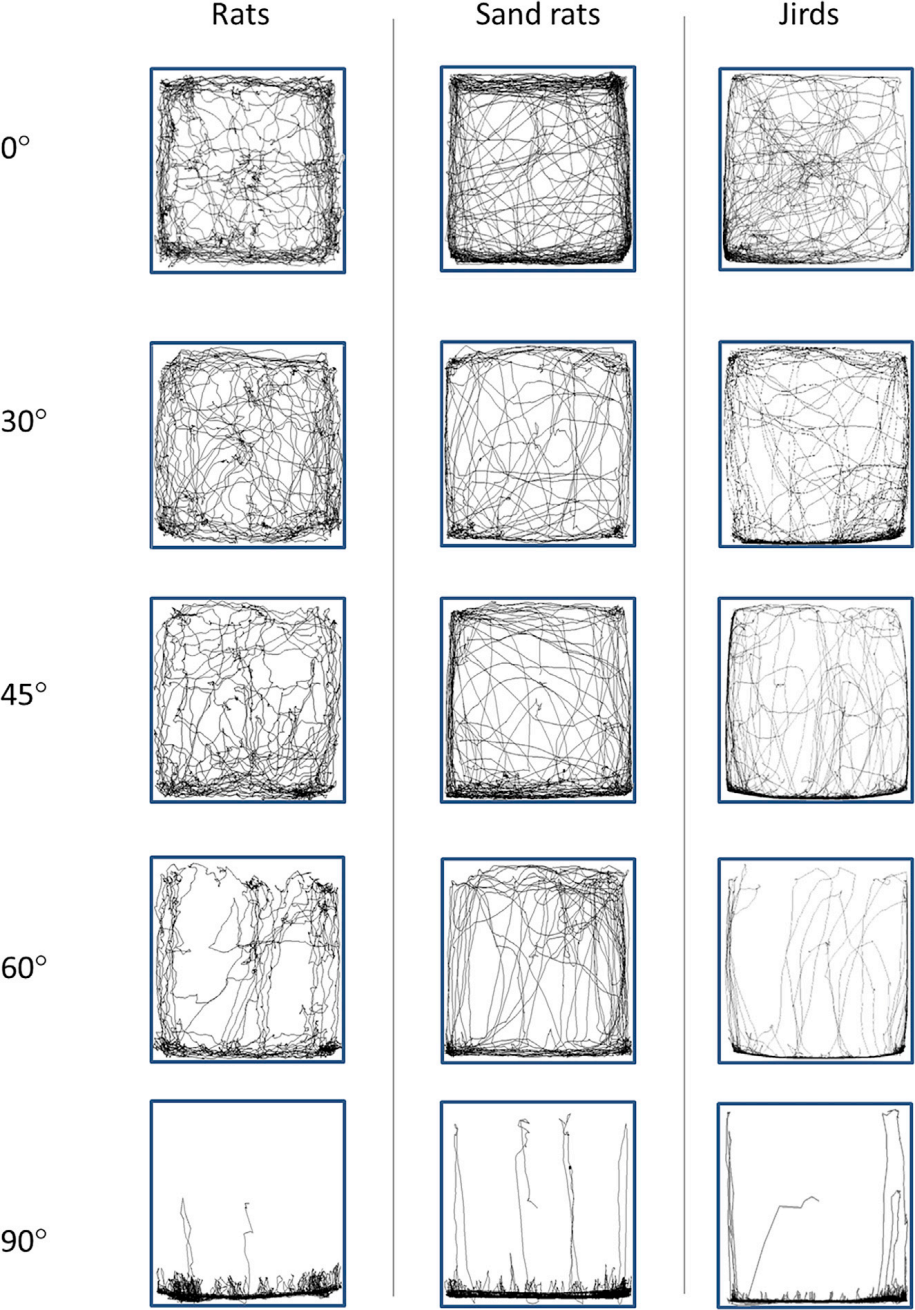
The tendency to restrict travel to along the bottom edge with the increase in open-field inclination. Trajectories of travel in the three species for each open-field inclination. Each inset depicts the trajectories of one rodent over the course of the 30 min test. The bottom face of each inset represents the lower edge of the open-field. As shown, while on the low inclinations, the rodents traveled throughout the open-field area, when the inclination rose to 45° or above, traveling became restricted mostly to a strip along the lower edge. At 90° inclination, they almost exclusively traveled along the lower edge, and their trajectories when away from the lower edge were typically vertical (straight up or down). Additional trajectories are available as supplemental materials Figures S2, S3, and S4.

**Figure 2:**
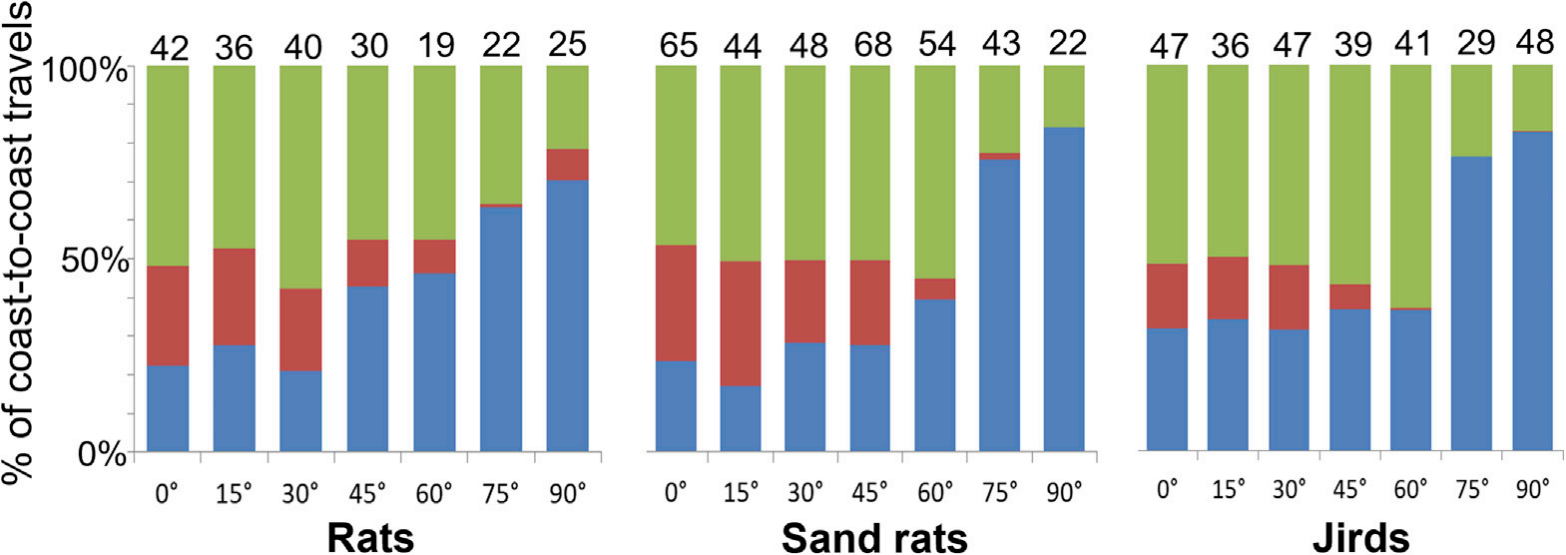
The preference to travel horizontally along the lower edge or vertically (straight up and down) with an increase in open-field inclination. For each species, the average number of horizontal and vertical coast-to-coast travels (from one wall of the open-field to the opposite wall) in each inclination, is depicted above of each bar. Horizontal travels are divided into those performed within the strip along the lower edge (), and those performed above it, within the rest of the open-field (). Vertical coast-to-coast travels are also depicted (). Because the rodents traveled throughout the area on the low inclination, the proportion of vertical travel and horizontal travel (in both the lower and upper parts) was about 50%–50% on inclinations up to 45°. On the higher open-field inclinations (60°, 75°, and 90°), traveling horizontally along the lower edge dominated, while elsewhere (away from the lower edge) it diminished to almost zero, and accordingly, traveling coast-to-coast was mainly horizontally along the lower edge or vertically up-down.

In addition to reducing their activity with the increase in inclination, all three species displayed an increasing tendency to spend a relatively large percentage of their travel time on the lower edge of the open-field (Figure 1). This was especially notable when traveling from one side of the open-field to the opposite side (“coast-to-coast”) (Figure 2). Because a large part of side-to-side traveling was along the lower edge, we also divided such travel into two parts: (i) in a strip along the lower edge and (ii) in the rest of the open-field. Vertical coast-to-coast travel is also depicted. As shown, on lower open-field inclinations (0°, 15°, 30°, and 45°), all species revealed approximately the same ratio of vertical and horizontal travel (both at the lower edge and in the center). On the higher open-field inclinations (60°, 75°, and 90°), traveling side-to-side along the lower edge dominated, while traveling side-to-side elsewhere (away from the lower edge) diminished to almost zero, and accordingly, was mainly along the lower edge and vertically up-down.

As shown in Figures 1 and 2, the distance traveled on the lower edge of the open-field was conspicuously greater than in any other direction of travel. Indeed, the metric distance traveled along the lower edge (15 cm strip) was significantly greater than the distance expected by the relative area of this strip out of the total open-field area. As shown in Table 2, in all three species, there was a significant effect of inclination, manifested as a trend of increased travel on the lower edge, and a significant interaction between inclination and the observed greater distance traveled on the lower 15 cm strip. Notably, on the lower 15 cm strip, the rodents could only travel horizontally (side-to-side) from left to right and vice versa. This greater proportion of travel on the lower strip masked any changes that could have taken place during traveling in the upper part of the open-field. In order to uncover such possible changes, we analyzed the rodents' behavior in the upper part of the open-field while excluding the lower 15 cm strip. In other words, while the three species tended to mainly travel horizontally on the lower strip, we also scrutinized how they traveled when away from this strip and on the upper part of the open-field.

**Table 2:** A strong preference to travel horizontally along the bottom edge of the open-field

Results of repeated measures ANOVA for each species					
		Distance (m.)	Effect of inclination	Expected vs. observed	Interaction: Inclination x expected-observed
Rats	expected	10.7 ± 0.5	F_6,63_ = 8.85	F_1,63_ = 234.24	F_6,63_ = 16.48
	observed	28.4 ± 1.9	**p < 0.0001** (η_p_^2^ = 0.4574)	**p < 0.0001** (η_p_^2^ = 0.7880)	**p < 0.0001** (η_p_^2^ = 0.6108)
Sand rats	expected	11.2 ± 0.7	F_6,63_ = 2.79	F_1,63_ = 140.81	F_6,63_ = 6.99
	observed	35.0 ± 2.9	**p = 0.0181** (η_p_^2^ = 0.2097)	**p < 0.0001** (η_p_^2^ = 0.6909)	**p < 0.0001** (η_p_^2^ = 0.3999)
Jirds	expected	11.1 ± 0.7	F_6,63_ = 2.31	F_1,63_ = 143.74	F_6,63_ = 4.85
	observed	41.2 ± 3.4	**p = 0.0448** (η_p_^2^ = 0.1800)	**p < 0.0001** (η_p_^2^ = 0.6953)	**p = 0.0004** (η_p_^2^ = 0.3158)

Mean ± SEM of the observed distance traveled (m.) in the bottom 15 cm zone on all open-field inclinations compared to the expected distance (15% out of the total distance traveled throughout the 1 × 1 m open-field area on all inclinations). Bold entries indicate a significance level of alpha < 0.05.

**Figure 3:**
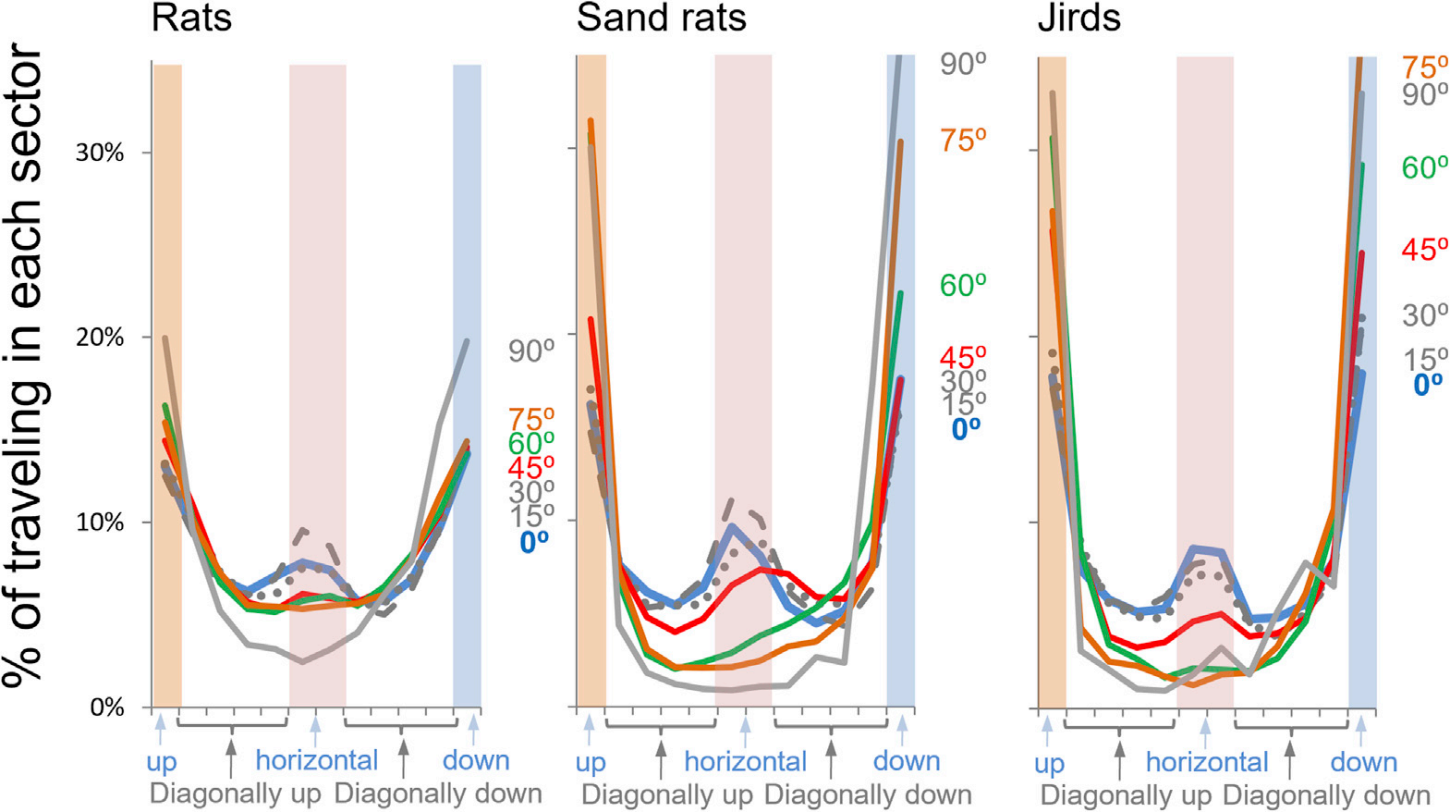
The strong preference to travel vertically straight up and down on an inclined surface (open-field) when away from the lower edge. For each rodent, data were extracted as follows. For each frame (0.04 s), we assumed that the rodent was standing in the center of a compass rose (in which 0 was up and 180 was down; see STAR Methods). The direction of progression of the rodent to the next frame was scored in sectors of 30° (the abscissa here is the compass rose described in STAR Methods). For each rodent, traveling in each direction (sector) was calculated as a percentage of the total frames (before the exclusion of traveling along the bottom edge, there were 45,000 data points (= frames)). Lines represent the average percentage for each direction in each open-field inclination; legends of inclinations are presented to the right according to the corresponding color for each species. As shown, all three species displayed a strong preference to travel straight up (orange shading) or straight down (blue shading). Altogether, about 70% of travel when away from the bottom edge was straight up or down. There was also an increased tendency to travel horizontally (pink shading). Clear areas in the figure represent diagonal travel up or down, which can be seen to have been mostly avoided and minimal, especially for the steep inclinations. While the above tendencies were similar in the three species, they were more conspicuous in the sand rats and jirds, and more subtle in the laboratory rats. Note that data in this figure represent only travel in the upper 85 cm of the open-field (away from the lower 15 cm strip, where the rodents traveled only horizontally).

To analyze the directions of travel in the upper part of the open-field, we assumed that in each frame (0.04 s) the rodent is standing at the center of a compass rose, and we scored its direction of progression in 30° sectors (see Figure S1C in supplementary information 1). Comparing left and right corresponding sectors revealed that the rodents did not display a bias for either left or right hemispheres, and we therefore combined the two hemispheres in further analyses (see STAR Methods). A repeated measure ANOVA with two factors (species and inclination) and 30° sectors (directions) as the within factor revealed that all factors had a significant effect (detailed in Table 3). Specifically, above the 15 cm lower strip, all three species displayed, with the increase in open-field inclination, a strong preference to travel up and down rather than horizontally or diagonally (Figure 3). This was especially prominent in the sand rats and jirds, for which about 70% of their travel outside the 15 cm lower strip was strictly up and down; this effect was also significant but more subtle in the rats. In addition, all three species displayed a significant decrease in traveling horizontally (side-to-side) outside the 15 cm bottom strip with the increase in open-field inclination (see also Figure 2 for the diminishing coast-to-coast horizontal travel outside the lower edge). Notably, for all inclinations, traveling diagonally up and down was always less than traveling either straight up/down or horizontally. Altogether, traveling underwent three major changes with the increase in open-field inclination: (i) a decrease in the traveled distance, (ii) an increase in traveling horizontally along the lower edge, and (iii) a marked preference to travel straight up and down when outside the lower edge. These three changes are apparent in the trajectories of representative individuals in each inclination for each species (see Figures S2, S3, S4 in supplementary information 2).

**Table 3:** The significant effect of inclination and species on the direction of travel in the upper part of the open-field (excluding the horizontal travel along the bottom edge)

Factors and interactions	F	P	η_p_^2^
Species	76.7 _[2,189]_	**< 0.0001**	0.4480
Inclination	68.3 _[6,189]_	**< 0.0001**	0.6843
Species x Inclination	9.3 _[12,189]_	**< 0.0001**	0.3719
Sector	664.1 _[11,2079]_	**< 0.0001**	0.7785
Sector x Species	33.4 _[22,2079]_	**< 0.0001**	0.2614
Sector x Inclination	20.9 _[66,2079]_	**< 0.0001**	0.3984
Sector x Species x Inclination	2.9 _[132,2079]_	**< 0.0001**	0.1563

Results of repeated measure ANOVA with two factors, Species and Inclination, and Sector (direction) as the within factor. The preference to travel vertically straight up and down is shown in Figure 3.Bold entries indicate a significance level of alpha < 0.05.

### Experiment 2

**Figure 4:**
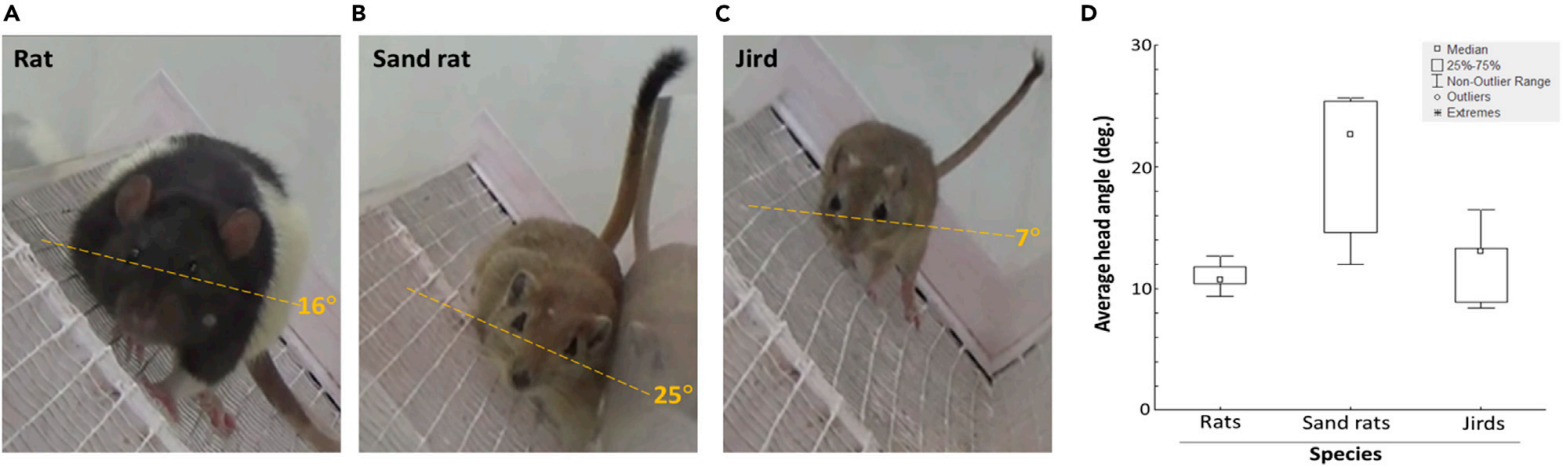
Rodents rotate their head to achieve a horizontal posture when traveling side-to-side on a 60° inclined surface. Photos of a rat (A), a sand rat (B), and a jird (C) while traveling side-to-side along a horizontal corridor with a wire-mesh floor inclined at 60° along its width. In each photo, the dashed line depicts the level of the eyes and their angle in relation to the horizontal axis. As shown, the angle of head rotation in sand rats was 2-fold that of rats and jirds (4D). Note that the sand rat travels while leaning against the corridor wall (4B), while the rat (4A) and jird (4C) travel in the center of the corridor.

In order to measure head posture during horizontal travel on a 60° inclined surface, rats, sand rats, and jirds were tested in a corridor (see Figure S1B in supplementary information 1), and the angle between head level and the horizontal axis was measured. Specifically, a line between the eyes represented head level (lateral head axis), and its angle in relation to the horizontal axis was measured (Figure 4). The average head angle of individuals (Figure 4D) was compared using one-way ANOVA, and revealed a significant difference between species (F_2,12_ = 6.93; p = 0.0099; η_p_^2^ = 0.5358). A Tukey HSD test revealed that head angle in sand rats was greater than in both rats and jirds. The videoclips of the rodents traveling in the corridor revealed the reason for the 2-fold greater head angle in sand rats: while the rats and jirds traveled on the 60° inclined surface away from the corridor walls, the sand rats traveled while leaning against the wall (see Figure 4B). Altogether, when traveling horizontally on a steep incline, all three rodent species rotated their head along its longitudinal axis to minimize the angle between head level (lateral head axis) and the horizontal axis.

## Discussion

**Figure 5:**
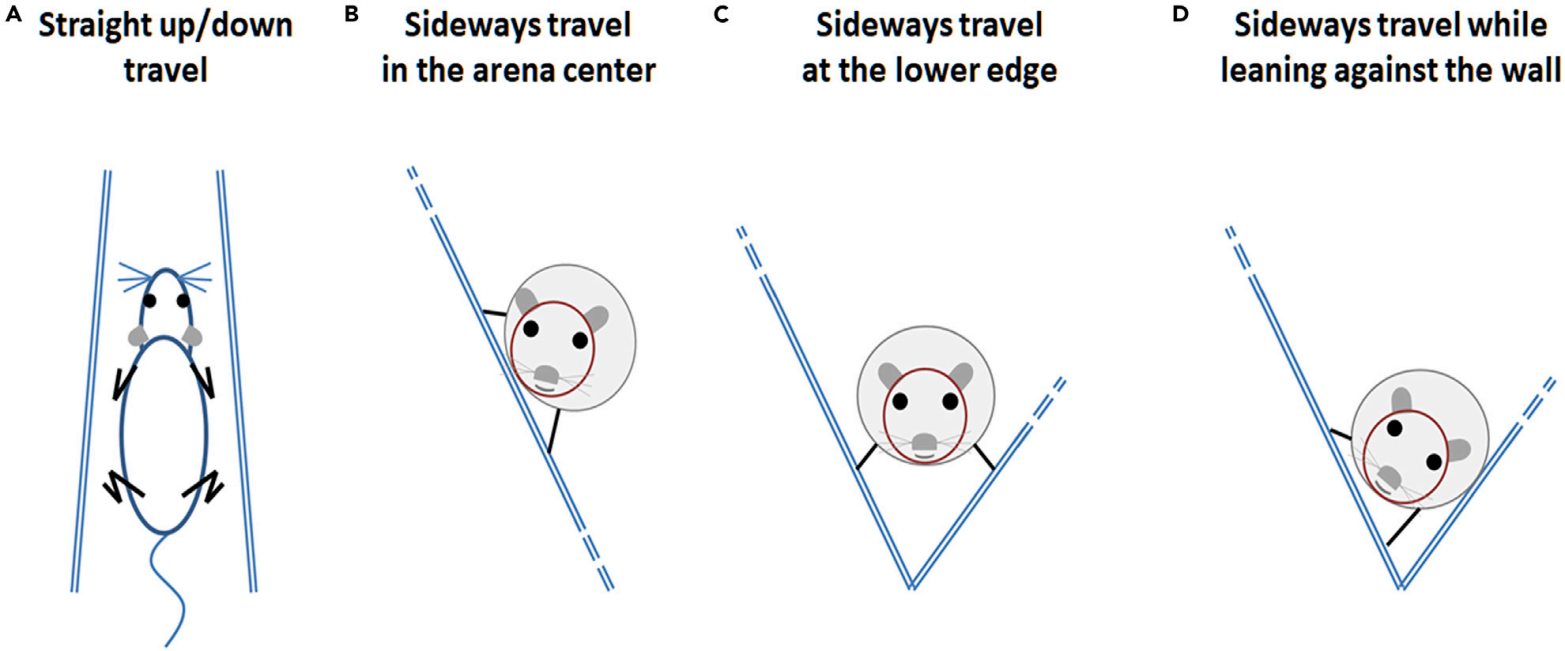
The four forms in which rodents kept a level head during traveling on an inclined surface. (A) When traveling vertically (straight up or down), the lateral axis of the head (represented by an imaginary line connecting the two eyes) was parallel to the horizontal plane. (B) When traveling horizontally on an inclined surface, they rotated their head along its longitudinal axis, thereby bringing the lateral axis of the head (eye level) near to the horizontal plane. (C) When traveling along the lower edge of the open-field next to the wall, the rodents walked with their legs on one side of the body stepping on the tilted floor of the open-field, and their legs in the other side stepping on the open-field wall. Accordingly, they were able to adopt a horizontal posture for both head and trunk. (D) The sand rats demonstrated another way of progression horizontally on a tilted surface by supporting their trunk against the wall. This provided them with stability and a postural reference that enabled them to progress with their head rotated at a relatively large angle that was not seen in the other rodents.

Surface-bounded animals may encounter various types of three-dimensional structures in their natural habitat, which may influence their spatiotemporal behavior and orientation. Here, we compared the behavior of rodents from three different habitats: sand rats, which forage on desert shrubs; jirds, which dwell in flatlands; and laboratory rats. These three species were tested in an open-field that was tilted at various inclinations. Despite the structural differences in their natural habitats, the behavioral changes displayed by the three species were the same: they all decreased their activity with the increase in open-field inclination. On the inclined open-field, they shifted to traveling mainly horizontally at the bottom or vertically (straight up and down) when away from the bottom. When traveling horizontally, they rotated their head to a nearly horizontal posture, while when traveling vertically straight up or down, the left and right sides of their head were maintained on the same level (Figure 5). Altogether, when we attempted to force the rodents to divert from the natural horizontal posture of their head and body, they overcame this challenge by the above modes of traveling. In the following discussion, we first suggest that the rodents' activity decreased with the increase in open-field inclination due to both physical and psychological load. We then conclude that since the above changes were observed to be similar in all three species regardless of their different habitats, maintaining a horizontal level of the head is a fundamental perceptual obligation when traveling, as can be seen when traveling in three-dimensional environments.

Compared to traveling in the horizontal domain (i.e. a surface perpendicular to gravity), animals that travel on non-horizontal surfaces require extra effort. For example, energy expenditure when traveling uphill is greater compared with traveling on a horizontal surface (Chavanelle et al., 2014; Minetti et al., 2002; Nardi et al., 2011). This was nicely demonstrated in rats that faced the choice of either ascending or descending in order to receive the same reward in each case. These rats displayed a preference for the downhill route to the reward, suggesting that this reflects a consideration of the lower energetic cost compared with the uphill route (Porter et al., 2019). Nonetheless, descending a slope too requires effort, in order to balance the body and avoiding sliding or falling, and this resulted in the rats traveling more slowly downhill than uphill (Porter et al., 2019). In addition to considerations of effort, descending seems to be more complex psychologically than ascending. Indeed, height was overestimated by humans when standing on a hilltop to a greater extent than when standing at the foot of the hill, and this difference was explained by the cost and the fear of falling, which are both greater in descending than in ascending (Jackson and Cormack, 2007; Okabe et al., 1986). Such factors could also account for the present finding that with the increase in inclination, the rodents decreased both their ascending and descending travel, preferring instead to travel horizontally, which at a 90° inclination became almost exclusive.

Different habitats may lead to different adaptations to the habitat’s physical and biological properties. These adaptations can be manifested as different motor capacities and different perceptions of the same environment (Gibson, 1979; von Uexkiill and O’Neil, 2010). Here, we found that all three rodent species displayed similar behavioral changes upon encountering an inclined surface compared to the organization of their spatial behavior on the horizontal plane. They all decreased their traveled distance, increased traveling horizontally along the lower edge, and traveled straight up and down, not diagonally, when outside the lower edge. Differences among the three species were only of degree and not of kind, and explicit in such a generality is that these changes are fundamental in spatial behavior.

When exploring an unfamiliar environment such as an open-field, the various visited locations are encoded in the brain in the order of their encounters, and then, through the processes of replaying and preplaying the encoded order, a mental image of the environment is acquired (Dragoi and Tonegawa, 2011). Head orientation plays an important role in generating a mental image of the environment, which then assists in orientating and navigating in that environment. For example, it was shown that head direction neurons encode the orientation of the head as an azimuth to a reference point (Laurens and Angelaki, 2018) in both the horizontal plane and in relation to gravity (Angelaki et al., 2020; Angelaki and Laurens, 2020). To this, we suggest that head alignment with the horizontal plane is also necessary when traveling and exploring the environment. Specifically, we found that when traveling on an inclined surface, the rodents attempted to maintain the frontal axis (left-right axis of their head; ear-to-ear, eye-to-eye) aligned with the horizontal plane. In other words, both eyes and both ears are held at the same horizontal level. To acquire this head posture, the rodents traveled horizontally while rotating their head along its longitudinal axis, or traveled vertically (straight up and down). In both these travel patterns, the frontal axis of the head was aligned with the horizontal plane. The question now arises as to what does an animal gain from maintaining the horizontal posture of the head frontal axis?

As noted in the “Introduction”, vestibular signals have an important role in spatial orientation and navigation (Banovetz et al., 2021; Goddard et al., 2008; Israel and Warren, 2005; Stackman et al., 2002), and disruption of vestibular activity affects neuron structure and activity in the hippocampus, interrupting the vestibular signals that are crucial for the activity of place cells and head direction cells (Harvey et al., 2018; Stackman et al., 2002; Yoder and Taube, 2014). Because disruption of the vestibular system severely affects spatial orientation (Brandt et al., 2005; Russell et al., 2003; Stackman et al., 2002), we suggest that adopting and maintaining a horizontal level of the frontal axis of the head, by traveling straight either horizontally (with the head rotated horizontally) or vertically, provides the animal with similar vestibular stimulation, which is essential for navigation and orientation.

**Figure 6:**
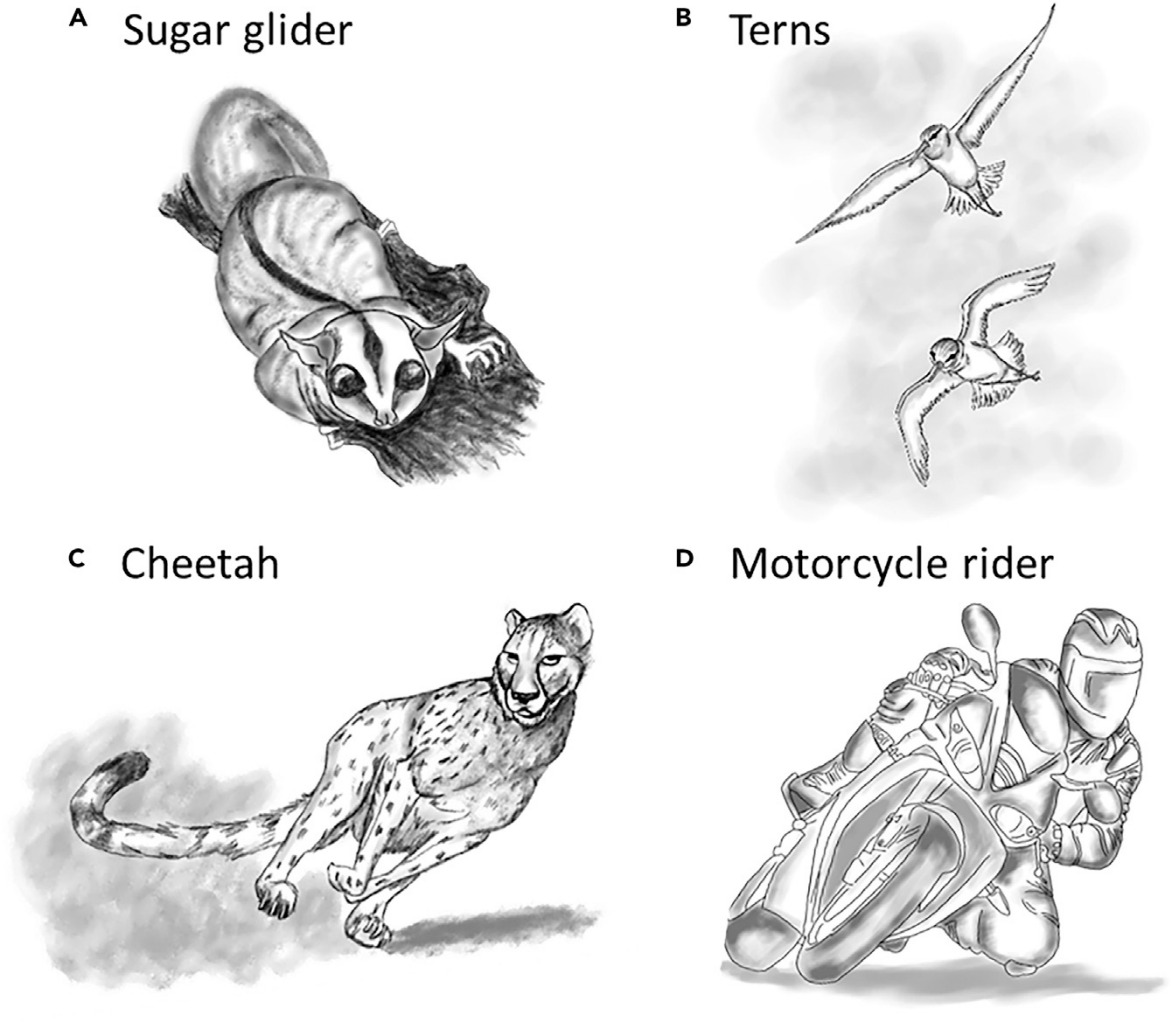
Keeping a level head in a human, mammals, and birds. Four different animals maintaining a level head in motion. (A) A sugar glider, an arboreal marsupial that travels on branches and regardless of body inclination maintains a level head posture. (B) Terns fly and adopt a level head posture while their trunk and wings are rotated in relation to the horizontal plane. (C). A cheetah maneuvering when chasing a prey maintains its head leveled in the horizontal plane. (D) A motorcycle rider during a sharp fast turn maintains a level head as otherwise he will lose orientation and crash. [figures drawn by Dr. Alex Dorfman based on photos].

Head stabilization is defined as “the ability to maintain an equilibrium orientation of the head with respect to space” (Cromwell, 2003). Among vertebrates, including humans, head stabilization is essential for spatial orientation, since stabilizing the head in relation to the horizontal plane increases the effectiveness of vestibular inputs (Zubair et al., 2016). The semicircular canals in the ears are sensory organs that send vestibular information to the brain and are therefore involved in head stabilization, as has been demonstrated in a variety of vertebrate species. For example, walking, trotting, and cantering horses align their horizontal semicircular canals to about 5° in relation to the horizontal surface (Dunbar et al., 2008). During walking, cats keep their head rotated up to 10° in relation to the horizontal surface, which is the sensitivity range of the horizontal semicircular canals for head orientation (Zubair et al., 2016). Humans rotate their head up to a maximum of 20° with respect to the horizontal plane during walking, running, and hopping (Pozzo et al., 1990). Similarly, pigeons horizontally stabilize their head during 90° hard turns (Ros and Biewener, 2017). When galloping, gray wolves and domestic dogs move their head up and down in the vertical domain but stabilize it with respect to the horizontal plane. In contrast, other cursorial mammals (e.g., cheetah) minimize the vertical movement of the head, which is stabilized both horizontally and vertically as if fixated in space and taking a straight trajectory (Wada, 2021). Interestingly, horizontal head stabilization in humanoid robots enables their accurate estimation of the gravitational force in the vertical domain, together with simplifying the dynamics of the entire robotic system (Farkhatdinov et al., 2011). Taken together, these studies demonstrate that head stabilization is based on maintaining a horizontal level of the frontal axis of the head, which is a basic requirement for spatial orientation and a salient feature of animals in motion. This is illustrated in Figure 6, which depicts four examples of vertebrates that keep a level head when traveling with their trunk rotated in relation to gravity. This is also what the rodents in the present study acquired when they traveled either horizontally with the head also rotated horizontally or straight up or down vertically, and therefore maintained a similar vestibular input to both ears.

### Limitations of the study

A drawback of the present study is that the rodents were tested in an illuminated environment with perceptible visual cues. While previous studies contending that visual cues do not compensate for a deficiency in vestibular cues support the present findings, running the same tests in the dark could explicitly highlight the vestibular impact. In the same vein, the application of a neurological vestibular approach (e.g. surgical or drug administration to the vestibular system) could highlight the underlying mechanisms that account for the behavioral results demonstrated in this study. Finally, it should also be noted that proprioceptive (Figures 4A, 4C, 5B and 5C) and/or tactile (Figures 4B and 5D) information can also be used to compensate for vestibular tilt.

## STAR★Methods

### Key resources table

**Table:** 

RESOURCE	SOURCE	IDENTIFIER
**Deposited data**		

Analyzed data	This paper	N/A

**Experimental models: Organisms**		

Long-Evans hooded rats	TAU animal quarters	N/A
Sand rats (*Psammomys obesus*)	TAU Research Zoo	N/A
Tristram’s Jirds (*Merions tritstrami*)	TAU Research Zoo	N/A

**Software and algorithms**		

Ethovision XT 11.5	Noldus	https://www.noldus.com/ethovision-xt
R	R foundation	https://www.r-project.org/
Reported experiment code	This paper	https://github.com/ZoharHTAU/Slope-terrain.git

### Resource availability

#### Lead contact

Further information and requests should be directed to and will be fulfilled by the lead contact, David Eilam (eilam@tauex.tau.ac.il).

#### Materials availability

This study did not generate new unique reagent.

### Experimental model and subject details

#### Experiment 1

##### Experimental design

The objective of this experiment was to test whether rodents modify their spatial behavior when traveling on a tilted platform compared with a horizontal one (open-field). For this, seven groups (n = 10 ea.) of each of the following three species were tested in only one of the following inclinations: 0°, 15°, 30°, 45°, 60°, 75°, and 90°. We compared the level of activity (traveled metric distance) and the distribution of activity as a function of the increased inclination.

The rationale for including three rodent species was to compare between species that are used to climbing up and down and species that live on a flat plane. Specifically, rats are a common experimental species and served here for comparing the present results with those of other studies. The laboratory rats were domesticated from Norway rats (*Rattus norvegicus*), which are large heavy-body commensal rodents that dwell in complex habitats. Sand rats (*Psammomy obesus*) were included since they forage by climbing on desert shrubs and feeding on their salty leaves. Jirds (*Meriones* *tristrami*) were included to represent animals that live in flatlands and are not accustomed to climbing up or down. Together, these three species represent life in a variety of three-dimensional environments.

##### Subjects

Seventy male Long-Evans hooded rats (weighing 200–400 g; age 3–6 months) were obtained from the animal quarters of the Faculty of Life Sciences at Tel-Aviv University and acclimated by handling for 10 min daily for two weeks prior to testing. Seventy male and female fat sand rats (weighing 196 g ± 4 g; age 3–6 months) and 70 male and female Tristram’s jirds (weighing 70 g ± 2 g; age 3–12 months) were taken from captive colonies from the I. Meier Segal’s Zoological Garden at Tel-Aviv University. Test groups of sand rats comprised five females and five males, and test groups of jirds comprised four females and six males on average. Despite the difference in sex composition among the groups, there was no difference in the level of activity between the three species (Table 1). All the rodents were maintained in a temperature-controlled room (23 ± 1 °C). For two weeks before testing, same-sex pairs of rats and jirds were housed in rodent cages (42 × 26.5 × 18.5 cm) with sawdust bedding, Fat sand rats were housed in rodent cages (56 × 39 × 28 cm; two to four animals per cage) with 12/12 dark/light phase; lights on at 6 a.m. since they are diurnal rodents. Standard rodent chow and fresh water were provided ad-lib for rats and jirds, while the fat sand rats received sugar-free rodent pellets. This study was carried out in strict accordance with the regulations and recommendations of the Institutional Committee for Animal Experimentation at Tel-Aviv University. This committee approved the protocols of this study (permits # 04-20-024; # 04-21-057; Head of the Ethics committee: Professor Ruth Shalgi).

#### Experiment 2

##### Experimental design

This experiment was performed following our observation that the rodents in Experiment 1 had rotated their head when traveling horizontally. Accordingly, this experiment was designed to measure the posture of the head during horizontal travel on a 60° tilted surface. The rationale was that at this angle and above, the rodents in Experiment 1 had displayed a significant deviation from their behavior at the lower angles. Rats, sand rats, and jirds were tested here in a corridor (see below) where they could only travel horizontally on an inclination, and the angle between their head level and the horizontal axis was measured.

##### Subjects

Male Long-Evans hooded rats (n = 5; weighing 200–400 g; age 3–6 months) were obtained from the animal quarters of the Faculty of Life Sciences at Tel-Aviv University and handled for 10 min daily for two weeks before testing. Male fat sand rats (*P. obesus*; n = 5; weighing 196 g ± 4 g; age 3–6 months), and male Tristram’s jirds (*M. tristrami*; n = 5; weighing 70 g ± 2 g; age 3–12 months) were obtained from captive colonies at the I. Meier Segal’s Zoological Garden at Tel-Aviv University. Due to the much smaller number of animals required in this experiment, we used here only males. In Experiment 1 we used both females and males due to the limited availability of rodents of the wild species.

### Method details

#### Experiment 1

##### Apparatus

The open-field was a 100 × 100 cm wire-mesh floor (1 × 1 cm grid) surrounded by 40 cm high transparent Plexiglas walls. The entire apparatus could be tilted at various angles (Figure S1A in supplementary information 1). A video camera (Ikegami B/W ICD-47E) located perpendicularly to the open-field floor provided a top view of the entire floor. The apparatus was illuminated with a dim light (1.31 Lux). Illumination also included two IR lights, which are invisible to the rodents but visible to the video camera and supported the sharp image required for tracking the animals. Testing was performed during the dark phase of the rats and jirds, which are nocturnal. Sand rats, which are diurnal rodents, were tested during their light phase under white light (950 Lux).

##### Procedure

Testing started with an individual rodent being introduced into the center of the open-field, facing the upper side of the apparatus, and its behavior was video-recorded for 30 min. At the end of the session the rodent was returned to its cage and the arena was mopped with soap and water to neutralize odors.

##### Data acquisition and analysis

The video file of each rodent was tracked using *EthoVision XT* 11.5 (*Noldus Information Technologies*, NL; Noldus et al., 2001) at a rate of 25 frames per second, and the X, Y, T coordinates were extracted automatically from the file. Data were then transferred to R software (version 4.0.2; R Core Team, 2020) to calculate the following parameters:

##### Distance traveled

The cumulative metric distance traveled in each inclination was calculated by R from the X-Y coordinates.

##### Coast-to-Coast travel

Traveling continuously from one side to the opposite side of the open-field. A distance of 20 cm or less between a wall and the center of the rodent mass was considered as being at the wall. This parameter was divided into three categories: a. horizontally along the lower edge - traveling side-to-side within a 15 cm strip at the bottom wall of the open-field; b. horizontally side-to-side anywhere outside the lower edge - traveling left-right or right-left with an average vertical shift of ca. 15 cm; c. vertical - traveling down-up or up-down with an average horizontal shift of ca. 15 cm.

##### Travel direction (upper open-field)

For each video frame (0.04 s), the direction of progression was scored in 30° sectors (Figure S1B in supplementary information 1). Rodents mostly traveled horizontally within the lower 15 cm strip, which masked the direction of their travel in the upper 85 cm of the open-field. We therefore separated the analysis of the directions of travel in the upper part of the open-field, excluding the lower 15 cm strip in which they could only travel horizontally. In other words, travel in all directions was possible only in the upper 85 cm zone of the open-field, and was analyzed as follows. In order to calculate the angle *α* of the direction of motion of the rodent relative to the Y axes, the dot product multiplication (Equation 1) was calculated. Here $\vec{v}=\left(\begin{array}{l} v_{x}\\ v_{y} \end{array}\right)$ is the rat motion vector, and $\vec{w}=\left(\begin{array}{l} 0\\ 1 \end{array}\right)$ is the unit y axes vector.

Equation 1 $$\cos\left(\alpha \right)=\frac{\vec{v_{1}}\cdot \vec{v_{2}}}{\left|\vec{v_{1}}\right|\cdot \left|\vec{v_{2}}\right|}$$

The distance traveled in each time step (one frame) was calculated as the size of the motion vector: $\left|\vec{v}\right|=\left|\left(\begin{array}{l} v_{x}\\ v_{y} \end{array}\right)\right|=\sqrt{v_{x}^{2}+v_{y}^{2}}$.

#### Experiment 2

##### Apparatus

The apparatus was a 100 cm long horizontal corridor, 20 cm wide, with a wire-mesh floor (1 × 1 cm grid) and 20 cm high opaque Plexiglas walls. The apparatus floor was tilted at an angle of 60° across the width of the corridor (Figure S1B). A video camera was located at one end of the corridor, providing a frontal view of the rodent when it traveled along the corridor toward the camera. The apparatus was illuminated with daylight (950 Lux) for all three species.

##### Procedure

A rodent was introduced into the corridor away from the camera and its behavior was video-recorded for 10 min (each rodent was tested only once). At the end of the session, the rodent was returned to its cage and the arena was mopped with soap and water to neutralize odors.

##### Data acquisition and analysis

For each sand rat and jird, 20 frames of a frontal view were captured and the angle between the lateral axis of the head and the horizontal level was measured.

##### Head posture

The lateral axis of the head (head level) was represented as a line connecting the two eyes or the base of the two ears. Rats were less active, and only nine to 22 frames of each animal were captured for measuring head level.

### Quantification and statistical analysis

#### Experiment 1

The level of activity (metric traveled distance) was analyzed with Factorial analysis of variance with two factors, species and inclination. The observed traveled distance along the bottom edge of the open-field was compared with the expected distance traveled in the same area. For this, we used repeated measure ANOVA for each species. The direction of traveling in the upper part of the open-filed was analyzed with a repeated measure ANOVA with two factors (species and inclination) and sector as the within factor. Alpha level was set to 0.05 in all the analyses.

#### Experiment 2

A one-way ANOVA was used to compare head level in the three species. Alpha was set to 0.05.

## Data Availability

• Data: All data reported in this paper will be shared by the lead contact upon request. Any additional information required to reanalyze the data reported in this paper is available from the lead contact upon request. • Code: Code for the reported experiment in this paper is publicly available on Github repository (Hagbi, 2022).
