# Supplementary material for: Keep a level head to know the way ahead: How rodents travel on inclined surfaces?
Source: iScience. 2022 May 18;25(6):104424. doi: 10.1016/j.isci.2022.104424 (PMC9157226; doi:10.1016/j.isci.2022.104424)

iScience, Volume *25*

Supplemental information

Keep a level head to know the way ahead: How rodents travel on inclined surfaces?

Zohar Hagbi, Elad Segev, and David Eilam

SUPPLEMENTARY INFORMATION 1 (refers to STAR METHODS)

Figure S1: The apparatus of Experiment 1 (a.), Experiment 2 (b.), and the compass rose (c.).

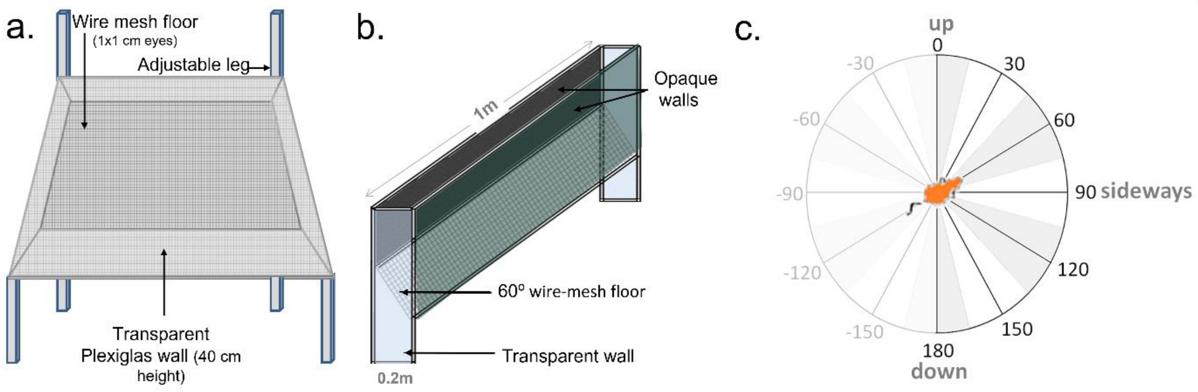

a. A 1 x1 m open-field with a wire-mesh floor with 1 cm holes, enclosed by a 40 cm high transparent Plexiglas wall. The walls were tilted inward to prevent the rodents from walking on them when the open-field was set to steep inclinations. At the rear ("upper") edge, there were long extendable legs that enabled the open-field to be inclined at any angle between 0° (horizontal surface) and 90° (vertical surface). A video camera was placed above at a perpendicular angle in relation to the open-field surface, providing a top-view of the apparatus.

b. A horizontal 1m long corridor with a wire-mesh floor (1 cm holes) inclined sideways at 60°. Two sides (lengths) of the corridor were enclosed by 20 cm-high walls. The other two sides (widths) were enclosed by transparent Plexiglass. A video camera placed at one end of the corridor provided a frontal view of the rodent when it traveled towards the camera.

c. The compass rose used to measure the direction of progression. The rose was divided at intervals of 30° degrees. Since the rodents did not display a left-right bias, we collapsed the two lateral sides of the rose, leaving only seven directions (0 = top; 30 = diagonally up; 60 = diagonally up-sideways; 90 = sideways; 120 = diagonally down-sideways; 150 = diagonally down-sideways; 180 = down). For each video frame (0.04 sec), a rodent was assumed to be standing at the center of the compass rose, and its direction of progression to the next frame was scored according to one of the above directions ± 15°. Therefore, each direction encompassed a 30° sector, as marked in the figure by shading (e.g., direction 0 was scored as 0° ± 15°). Notably, this scoring was performed only for travel away from the 15 cm strip along the bottom edge, where the rodents could travel only horizontally. The compass rose directions, from top to bottom, are the abscissa in Figure 3.

SUPPLEMENTARY INFORMATION 2 (refers to figure 1)

Figure S2: Trajectories of travel of four rats at each inclination (in addition to that shown in Figure 1). As shown, the rats traveled all over the open-field area on the shallow inclinations, but with the increase in inclination they switched to traveling mainly along the bottom edge or vertically straight up and down.

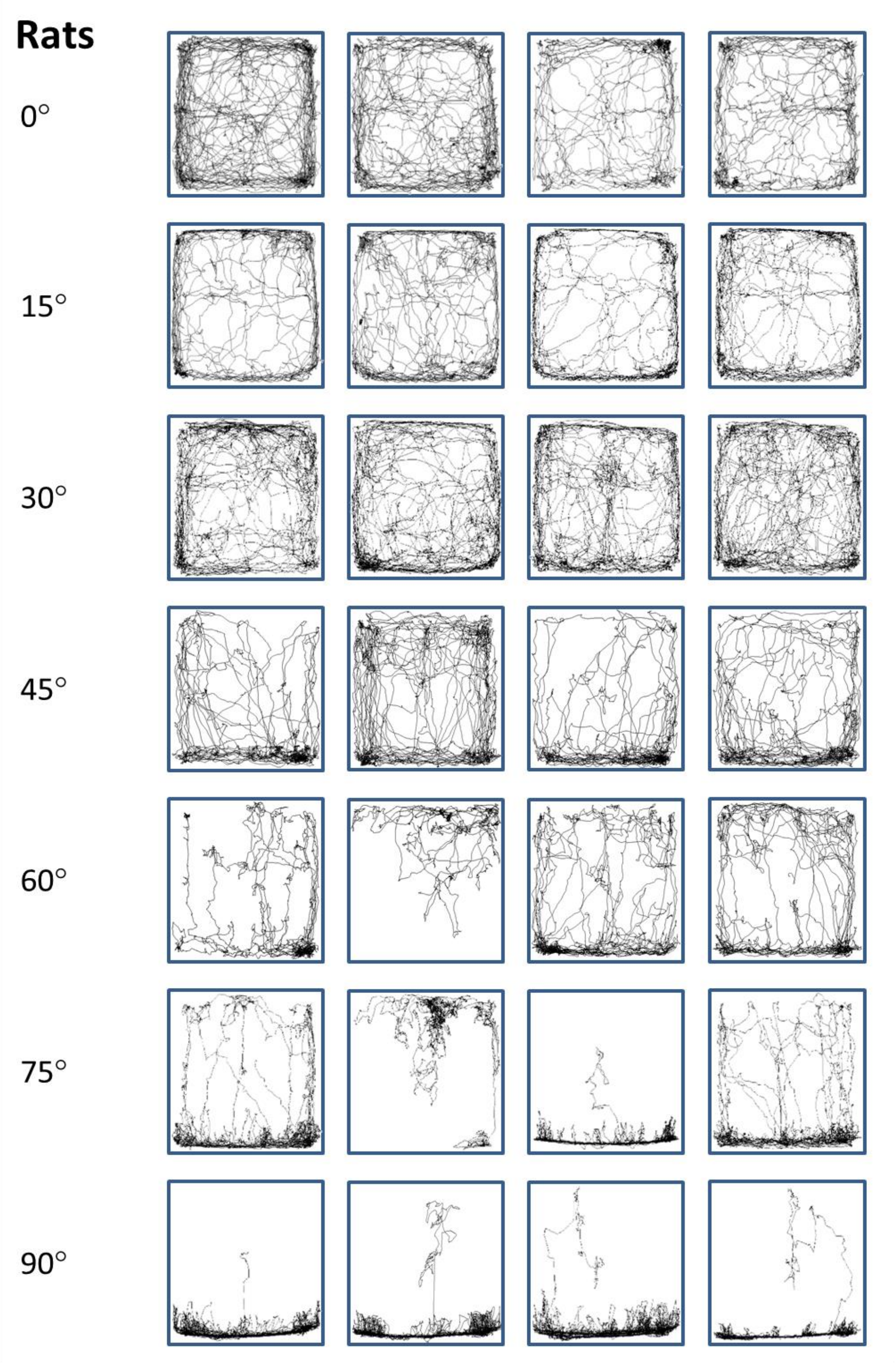

Figure S3: Trajectories of travel of four sand rats on each inclination (in addition to that shown in Figure 1). As shown, they traveled all over the open-field area on the shallow inclinations, but with the increase in inclination they switched to traveling mainly along the bottom edge or vertically straight up and down.

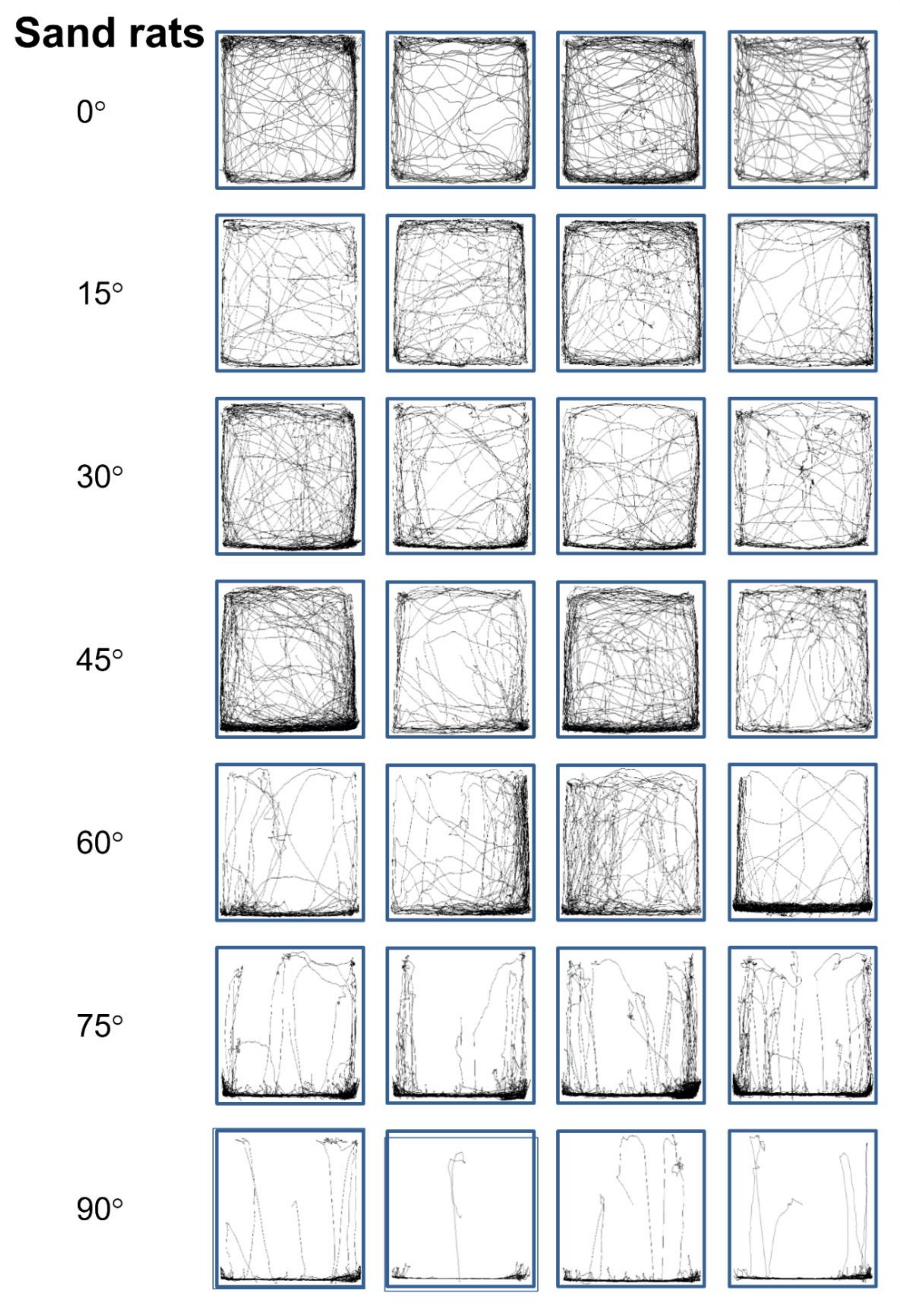

Figure S4: Trajectories of travel of four jirds on each inclination (in addition to that shown in Figure 1). As shown, they traveled all over the open-field area on the shallow inclinations, but with the increase in inclination they switched to traveling mainly along the bottom edge or vertically straight up and down.

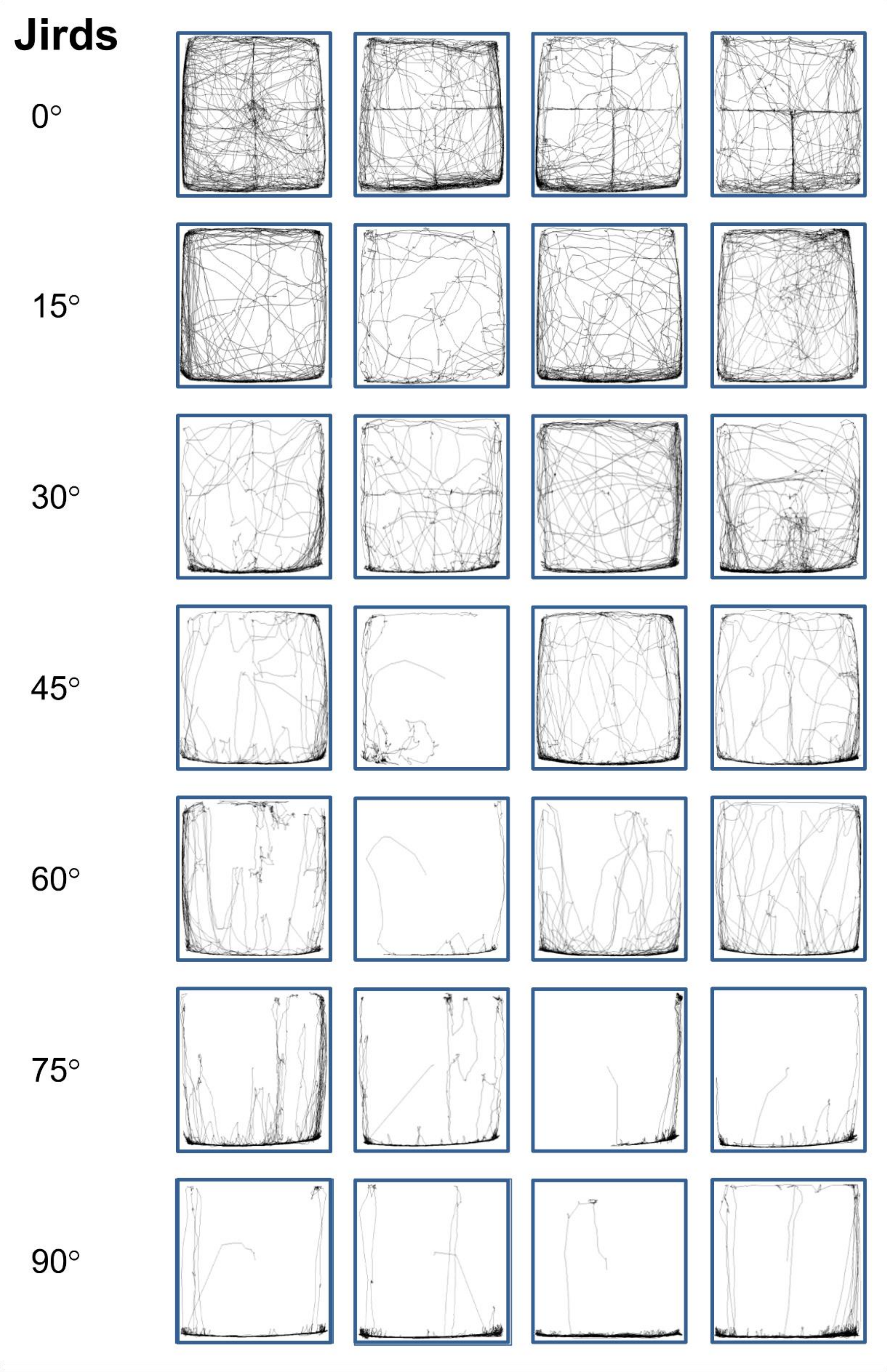

Supplement: Document S1. Figures S1–S4 [file mmc1.pdf]
